# Technical Implementation of a Multi-Component, Text Message–Based Intervention for Persons Living with HIV

**DOI:** 10.2196/resprot.2017

**Published:** 2012-11-16

**Authors:** Robert D Furberg, Jennifer D Uhrig, Carla M Bann, Megan A Lewis, Jennie L Harris, Peyton Williams, Curtis Coomes, Nicole Martin, Lisa Kuhns

**Affiliations:** 1 RTI InternationalRTP, NCUnited States; 2 Howard Brown Health CenterChicago, ILUnited States

**Keywords:** short message service, SMS, text message, mobile phone, mHealth, HIV, tailored messaging

## Abstract

**Background:**

Men who have sex with men (MSM) continue to be severely and disproportionately affected by the HIV/AIDS (human immunodeficiency virus/acquired immune deficiency syndrome) epidemic in the United States. Effective antiretroviral therapy has altered the HIV epidemic from being an acute disease to a chronic, manageable condition for many people living with HIV. The pervasiveness, low cost, and convenience of Short Message Service (SMS) suggests its potential suitability for supporting the treatment of conditions that must be managed over an extended period.

**Objective:**

The purpose of this proof-of-concept study was to develop, implement, and test a tailored SMS-based intervention for HIV-positive MSM. Prior studies do not routinely provide sufficiently detailed descriptions of their technical implementations, restricting the ability of subsequent efforts to reproduce successful interventions. This article attempts to fill this gap by providing a detailed description of the implementation of an SMS-based intervention to provide tailored health communication messages for HIV-positive MSM.

**Methods:**

We used archives from the SMS system, including participant responses to messages and questions sent via SMS, as the data sources for results reported in this article. Consistent with the purpose of this article, our analysis was limited to basic descriptive statistics, including frequency distributions, means and standard deviations.

**Results:**

During the implementation period, we sent a total of 7,194 messages to study participants, received 705 SMS responses to our two-way SMS questions of participants, and 317 unprompted SMS message acknowledgements from participants. Ninety two percent of participants on antiretroviral therapy (ART) responded to at least one of the weekly medication adherence questions administered via SMS, and 27% of those had their medication adherence messages changed over the course of the study based on their answers to the weekly questions. Participants who responded to items administered via SMS to assess satisfaction with and use of the messages reported generally positive perceptions, although response rates were low overall.

**Conclusions:**

Results confirm the technical feasibility of deploying a dynamically tailored, SMS-based intervention designed to provide ongoing behavioral reinforcement for HIV-positive MSM. Lessons learned related to text programming, message delivery and study logistics will be helpful to others planning and implementing similar interventions.

## Introduction

The annual number of newly diagnosed human immunodeficiency virus (HIV) infections has remained relatively stable in the United States since the late 1990s, with more than 50,000 people becoming infected annually [1]. Meanwhile, HIV prevalence in the United States is higher than it has ever been, with more than an estimated 1 million adults and adolescents living with HIV in the United States [2]. Men who have sex with men (MSM) continue to be disproportionately affected by the HIV/acquired immune deficiency syndrome (AIDS) epidemic, accounting for just over half of all new infections in 2006 [1]. Because of advances in antiretroviral therapy, people are living with HIV for longer periods of time [3]. For people living with HIV, antiretroviral therapy has transformed HIV into a chronic health condition that can be managed via medication adherence [4]. However, adopting and maintaining healthy behaviors and a medication regimen over a lifetime is challenging and may require ongoing behavioral reinforcement [5].

Given that among American adults, more than 80% own a mobile phone [6], with no significant differences in ownership by race/ethnicity [7], health-related text messages delivered to mobile phones could have a broad reach. As of December 2010, more than 302 million wireless subscriptions were active in the United States, and these domestic users sent and received more than 187 billion text messages each month [8].

The pervasiveness, low cost, and convenience of Short Message Service (SMS, or text messaging) suggests that it may be a channel particularly well suited for supporting the treatment of diseases or conditions that must be managed over an extended period [9,10]. SMS not only facilitates more frequent communication with patients but also offers the opportunity to deliver health-related messages when and where these messages may have the greatest impact, such as medication reminders consistent with an individual’s dosing schedule. Therefore, SMS may be one channel by which to provide ongoing reinforcement for people living with HIV related to medication adherence and maintenance of healthy behaviors. If effective, such an intervention may result in higher health care quality and better outcomes.

Despite the potential of SMS interventions delivered via mobile phone, a review of the literature on SMS-based interventions revealed that most prior studies have not included detailed descriptions of their technical implementation processes [11]. This gap is important because a systematic description of the technical implementation and message delivery could inform the design of future studies and strengthen the evidence base of this emerging research area. We sought to address this gap by documenting the technical implementation of our intervention. In this paper, we address the following questions:

1. How was the intervention implemented from a technical standpoint?
2. What changes to the technical implementation were made during the study?
3. Did participants respond to the messages and/or questions administered via SMS?
4. What were the participants’ perceptions of the content, timing, volume, usefulness, and helpfulness of the messages?

## Methods

### Study Design

**Figure 1:**
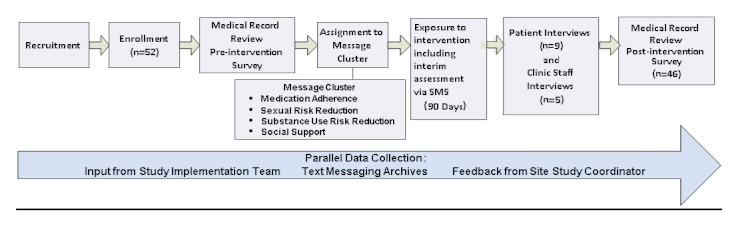
Study Design.

RTI International, an independent nonprofit research organization, partnered with Howard Brown Health Center (HBHC), an ambulatory care clinic in Chicago, to implement and evaluate an SMS-based intervention for HIV positive MSM to enhance outcomes related to managing HIV. RTI also partnered with Intelecare, a company that specializes in personal notification and communication management for medication adherence and disease management, to provide the two-way text messaging gateway. The SMS platform was used not only to deliver the messaging intervention, but also as a mode of primary data collection on weekly self-reported medication adherence, sexual risk and substance use risk behaviors at 30 and 60 days, and participant satisfaction intermittently throughout the 90-day intervention period. Our study design (see Figure 1) was reviewed and approved by the Institutional Review Boards (IRBs) at both RTI and HBHC. Each participant also signed an authorization for use or disclosure of health information to be compliant with the Health Insurance Portability and Accountability Act.

Eligible participants included English-speaking, HIV-positive MSM aged 25 and older who had personal cell phones, agreed to allow us to access their medical records, and were amenable to receiving SMS messages during the 3month intervention. HBHC providers identified eligible participants during routine visits for primary care; other participants self-referred to the study after seeing posters or flyers in the clinic’s waiting or examination rooms.

During initial screening, we confirmed eligibility and documented the participant’s cell phone number and ability to send and receive text messages. Next, we administered informed consent. During the informed consent process, we used a message tailoring form to document each participant’s preferences for receiving certain types of messages during the study or declining receipt of certain categories of messages, as required by RTI’s IRB. After obtaining informed consent, we assigned each participant a personal identification number (PIN), which served to anonymize the information required to carry out the intervention as well as each participant’s evaluation data. We entered the participant’s PIN, cell phone number, and message cluster assignment in the SMS gateway manager. Next, we administered a comprehensive Web-based preintervention assessment survey to each participant at the clinic. We used these data to tailor specific message content for the 3-month intervention. To minimize potential attrition from loss of cell phone service, we provided each participant with a $25 incentive upon enrollment and $10 per month for the 3-month study period to offset the costs associated with monthly SMS plans.

### Intervention Development

We developed the intervention implemented in our study based on a conceptual model developed by Coomes and colleagues [11]. The model integrates the communication functionality of SMS with important psychosocial factors that could mediate the impact of SMS communication on health care quality and outcomes. We developed the actual message content by beginning with a set of draft HIV prevention messages previously developed for HIV-positive individuals using a systematic formative research process [12]. We mapped each of the existing messages to the topic areas included in the current intervention (medication adherence, sexual risk reduction, substance use risk reduction, smoking cessation, general health and well-being, social support and patient involvement), identified gaps, and developed additional messages to fill those gaps. Three experts external to the project team, as well as health care providers at HBHC, reviewed and provided feedback on the draft messages. Finally, we qualitatively pretested the messages using in-depth, one-on-one interviews with 8 members of the target audience prior to disseminating them as part of the study.

We addressed each of the 8 topics below:

*Medication adherence*. Participants who reported a history of medication nonadherence in the week before the baseline assessment or who began antiretroviral therapy within the past 6 months received daily messages designed to complement their prescribed (Rx) regimens (e.g., “It’s going to be a great day. This is your med reminder.”). For example, patient-customized notifications were used to remind participants to take their medications, consistent with their clinical dosing schedule, which is generally 1–3 times per day. Participants who were therapeutically adherent received weekly adherence messages that encouraged them to continue taking their medications as prescribed (e.g, “He shoots! He scores! Perfect med adherence. Great job!). If at any point during the intervention therapeutically adherent patients reported missing any doses of their medication, adherence reminders were enabled and these participants received adherence reminders for the remainder of the messaging exposure.

*Sexual risk reduction*. Participants who reported at least one sex partner in the past 3 months received messages designed to reinforce condom use and communication with sex partners about HIV and other sexually transmitted infections (e.g., “Undetectable is respectable, but your partners are still infectable. Play safe.”). Messages regarding sexual risk reduction were sent on Saturday evenings. Sexual risk reduction was reassessed against baseline on intervention days 36 and 64 via SMS. Participants who reported at least one sex partner in the past 3 months triggered the dynamic tailoring function and those individuals began receiving the sexual risk reduction content for the remainder of the messaging exposure.

*Substance use risk reduction*. Considering substance use behaviors within the past 3 months, participants who reported drinking an alcoholic beverage2or more times per month, having 5or more drinks within a couple of hours at least once in the past month, or using any illicit drugs received messages to reduce use or harm (e.g., “Going out tonight? Be safe. Party smart.”). Messages regarding substance use risk reduction were sent on Friday evenings. Substance use risk reduction was reassessed against baseline on intervention days 36 and 64 via SMS. Participants who reported a history of any high-risk substance use triggered the dynamic tailoring function and those individuals began receiving the substance use risk reduction content for the remainder of the messaging exposure.

*Sexual and substance use risk reduction*. Because risky sex and substance use often co-occur, we developed a separate set of messages for Saturday evening delivery. Participants who reported any co-occurrence of substance use and sexual activity within the past 3 months received, in addition to the standard risk reduction messages, communications designed to reinforce the risk reduction messages and reduce harm (e.g., “No condoms? No way! Party n play the right way. Protect yourself and your partner.”). Sexual and substance use risk was reassessed against baseline on intervention days 36 and 64 via SMS. Participants who reported a history of substance use before or during sex triggered the dynamic tailoring function and those individuals began receiving the sex and substance use risk reduction content for the remainder of the messaging exposure.

*Cigarette smoking*. Because smoking cigarettes weakens the immune system, it can be especially harmful to persons with HIV. We included smoking cessation messages for participants who reported smoking (e.g, “There are many ways to quit smoking. Talk to your HBHC provider about the ways that would work best for you.”). Smoking cessation messages were sent every Thursday.

*General health and well-being*. We anticipated that all study participants would benefit from periodic messages emphasizing the value of a healthy lifestyle (e.g., “Take care of yourself today. Eat healthy foods, don’t stress out, get some exercise and sleep well.”). These messages were delivered to all participants every Wednesday.

*Social support*. In addition to general social support messages delivered every Sunday (e.g., “Worried about telling your friends and family your status? We can help you find the right words. Call HB at XXX-XXX-XXXX.”), we cataloged available social support resources sponsored by HBHC. These include HIV support groups, substance abuse groups, health and well-being forums, and other meetings and events of interest. On the basis of participants’ demographic and preintervention assessment survey responses, we notified participants of relevant support groups, meeting schedules and contact information for joining.

*Patient involvement*. All participants received messages aimed to empower themselves to be active health care consumers (e.g., “Ask your provider questions. If you don’t understand the answer, keep asking until you do.”). These messages were delivered to all participants on Mondays.

*Appointment reminders*. All participants received clinical and behavioral health appointment reminders as part of the intervention. A single appointment reminder was sent to participants at a randomly selected time within a 3 day window prior to scheduled appointments.

### Data Sources

We used archives from the SMS system, including participant responses to the messages and questions sent out via SMS, as the data sources for results reported in this paper. We have reported detailed descriptions of the data sources, methodology and results from the process and outcome evaluations elsewhere [13, 14, 15].

A unique feature of our design was the use of bidirectional messaging to support interactions with study participants and to allow us to dynamically tailor content throughout the intervention. We asked questions of all participants throughout their exposure to the intervention, and their responses were used to update the content they received, when appropriate. We administered 3 types of questions via two-way SMS: weekly medication adherence assessment, participant satisfaction items, and sexual and substance use risk reduction reassessment.

#### Weekly Medication Adherence Assessment

Every Sunday evening, we asked participants if they have missed any antiretroviral therapy doses in the preceding week by sending the SMS message: “Over the past 7 days, on how many days did you miss a dose of medication? Please text us back the number of days you missed a dose (0–7).” We processed responses to determine whether participants had been adherent to their regimen. Every Monday, we sent participants the appropriate feedback responses based on their answers. Specifically, adherent participants received a supportive response to continue, and nonadherent participants received encouragement to comply with their regimen in the week ahead. If at any time during the program a previously adherent participant reported a missed dose, we began sending him daily medication reminders tailored to his dosing schedule for the duration of the intervention.

#### Interim Participant Satisfaction

We asked all participants 8 questions to assess participant satisfaction with the intervention, including feedback on the frequency of messaging and the relevance of content (see Table 1). In an effort to distribute the response burden, we sent2questions per week throughout Weeks 6–9. To manage incoming messages from participants and differentiate responses to the satisfaction questions from the sexual and substance risk assessment questions, we instructed subjects to provide responses that included number and letter combinations that corresponded to specific questions, as shown in Textbox 1.

**Participant Satisfaction Items (delivery Via 2-Way Sms)**

How often do you read the text messages you get from HB?

Text 1A = always, 2A = usually, 3A = sometimes, 4A = never

Do you like the messages you are receiving from HB^a^?

Text 1B = yes, 2B = no

How often are the HB messages sent at the right times?

Text 1C = always, 2C = usually, 3C = sometimes, 4C = never

How do you feel about the number of text messages you get from HB?

Text 1D = too many, 2D = about right, 3D = not enough

Are the message topics you get from HB interesting to you?

Text 1E = very, 2E = somewhat, 3E = a little, 4E = not at all

How often do you use the info in the text messages from HB?

Text 1F= always, 2F = usually, 3F = sometimes, 4F = never

How helpful are the text messages you get from HB?

Text 1G = very, 2G = somewhat, 3G = a little, 4G = not at all

Do you feel like the HB messages were written for you?

Text 1H = yes, 2H = no

#### Sexual and Substance Use Risk Reduction Reassessment

We reassessed risk-taking behaviors related to sexual activity and substance use for all participants on days 36 and 64 of the intervention (see Textbox 2).

“Yes” and “don’t remember” responses were analyzed and used to initiate sending risk-reduction messages to those individuals who did not initially qualify for receiving messages in these categories.

**Sexual and Substance Use Reassessment Items (delivery Via 2-Way Sms)**

In the past 4 weeks have you had 5 or more drinks of alcohol within a couple of hours (e.g. 2–4 hours)?

Text 1i = yes, 2i = no, 3i = don’t remember

In the past 4 weeks have you used recreational drugs (e.g., pot, meth, cocaine or heroin)?

Text yes = 1J, 2J = no, 3J = don’t remember

In the past 4 weeks have you had sex without a condom with any of your sex partner(s)?

Text 1K = yes, 2K = no, 3K = don’t remember

In the past 4 weeks have you used alcohol or drugs before or during sex?

Text 1L = yes, 2L = no, 3L = don’t remember

### Analytic Methods

#### Messages Sent to Participants

To determine message intensity, we classified the timing of the messages on the basis of each participant’s week of participation in the study, using the date the first study message was sent to the participant as the start of his study participation. We then computed the mean number of messages participants received during each of the 13 study weeks using the SAS statistical software program.

#### Messages Received From Participants

For each of the questions administered via SMS, except medication adherence, participants provided a number and letter response (e.g., 1D) to indicate the question to which they were responding and the appropriate response option. Responses to the medication adherence questions consisted of a number from 0 to 7. On the basis of these codes, we classified participants’ text responses into the following 5categories, using the SAS software program to search the texting data for appropriate responses (1) responses to participant satisfaction questions (e.g., how helpful texts are), (2) reassessments of medication adherence and sexual and substance use behaviors, (3) acknowledgments from the participant that he received the message (e.g., “OK,” “Thanks”), (4) requests to stop receiving messages, and (5) responses that did not fit into any of the other 4 categories. Given the possible differences in texting style (e.g., using zero for O, using abbreviations, leaving out spaces), we reviewed the data manually to ensure that responses were placed into the appropriate categories—for example, to ensure that a response of “2doses” was not inadvertently counted as a “2d” response to a participant satisfaction question. In the event that a participant responded more than once to the same question, we used his last response. Finally, we computed the percentages of respondents indicating each response option.

## Results

### Message Delivery Schedule

The complexity of our intervention required us to design a flexible approach to tailoring the messages sent to each participant. To manage each subject’s preferences for which messages they were willing to receive, we parsed the core content into 24 message classes for processing and programming purposes (see Table 1).

Table 2 illustrates the message delivery schedule by topic and by day for the 13-week SMS intervention.

### Tailoring Process

We enrolled 52 participants over a 4-month period (July-October 2010). We enrolled new participants into the study every Friday throughout the 4-month recruitment phase. At the time of enrollment, we used data from the HBHC-administered screener, the message tailoring form, and the preintervention survey to create a unique text message profile for each subject. Our tailoring logic, including questions and responses that assigned participants into each message class is shown in Table 3. We used a tailoring form to code the critical values from these 3 data sources into an electronic tailoring worksheet and to manually create each participant profile.

Message content, tagged by class and day of intervention, comprised the technical script for this 90-day, automated intervention. The most significant effort in preparing for the implementation was to establish common vocabulary and a series of processes and documentation to support data exchange between RTI and Intelecare.

Both incoming and outgoing data from RTI were formatted based on the output of the tailoring process and converted to an eXtensible Markup Language (XML) “UserList” that includes the items listed in Table 4. The UserList was posted by 10:00 p.m. every Saturday throughout the implementation phase. To facilitate the transfer of the UserList, a File Transfer Protocol (FTP) site that utilized a 256-bit Advanced Encryption Standard connection was used to post the data directly to the SMS gateway. All messages were sent with the preface “<HB>” (short for “Howard Brown Health Center”) so participants could identify that the messages were coming from the study.

**Table 1:** Message class architecture

Class Number	Text Name	Notes	Time to Send
1	Weekly adherence question	Weekly on Sunday.	17:00
		All participants will be set up to receive this when their accounts are created.	
2	“Took all” response to #1	Will come in with Monday data.	16:00
		1 message for following Tuesday.	
		Message depends on place in cycle.	
3	“Missed” response to #1	Will come in Monday data.	16:00
		1 message for following Tuesday.	
		Message depends on place in cycle.	
4	Rx daily adherence	Different message each day for 7 days.	15:00
		Repeats weekly throughout program.	
		A single custom message is optional.	
		A custom time can also be defined by the participant.	
5	Rx am adherence	Different message each day for 7 days.	08:00
		Repeats weekly throughout program.	
		A single custom message is optional.	
		A custom time can also be defined.	
6	Rx pm adherence	Different message each day for 7 days.	21:00
		Repeats weekly throughout program.	
		A single custom message is optional.	
		A custom time can also be defined.	
7	Sex risk	Different message every Saturday.	22:00
8	Substance risk	Different message every Friday.	22:00
9	Sex & substance risk	Different message every Saturday.	22:00
10	Smoking	Different message every other Thursday, starting second Thursday.	10:30
11	General health & wellness	Different message every Wednesday.	10:00
		All participants will be set up to receive this when their accounts are created.	
12	General social support	Different message every Sunday.	14:00
		All participants will be set up to receive this when their accounts are created.	
13	Patient involvement	Different message every Monday.	09:30
		All participants will be set up to receive this when their accounts are created.	
14	Process question	Different questions days 38, 40, 45, 47, 52, 54, 59, & 61.	12:30
		All participants will be set up to receive this when their accounts are created.	
15	Substance question	Question at days 36 & 64.	10:30
16	Sex question	Question at days 36 & 64.	11:30
17	Substance & sex question	Question at days 36 & 64.	12:30
18	General social support	Different messages days 9, 10, 11, & 12.	12:30
19	Tailored social support 1	Message delivered day 13.	12:30
20	Tailored social support 2	Different messages days 14 & 15.	12:30
21	Tailored social support 3	Message delivered day 16.	12:30
22	Tailored social support 4	Message delivered day 17.	12:30
23	Tailored social support 5	Message delivered day 18.	12:30
24	Tailored social support 6	Message delivered day 19.	12:30

**Table 2:** Message delivery schedule by topic

Topic	Sunday	Monday	Tuesday	Wednesday	Thursday	Friday	Saturday
Medication reminder	X	X	X	X	X	X	X
Appointment reminders	PRN^a^	PRN^a^	PRN^a^	PRN^a^	PRN^a^	PRN^a^	PRN^a^
Rx adherence assessment	X						
Rx adherence response		X					
Patient involvement		X					
Health and wellness				X			
Smoking cessation					X		
Substance risk						X	
Sexual risk							X
Sex & substance risk							X
Risk questions	Days 36 & 64						
Participant satisfaction questions			Weeks 6–9		Weeks 6–9		
Tailored social support		Days 9–19	Days 9–19	Days 9–19	Days 9–19	Days 9–19	

^a^PRN, as needed.

**Table 3:** Tailoring logic

Message Class	Topic	Question	Response Options	Question	Response Options	Question	Response Options
2	Response: adherent	Many people don’t take their HIV medication perfectly all the time. Over the past 7 days, on how many days did you miss a dose of your HIV medication?	0				
3	Response: nonadherent	Many people don’t take their HIV medication perfectly all the time. Over the past 7 days, on how many days did you miss a dose of your HIV medication?	1, 2, 3, 4, 5, 6, 7, Don’t know, Refused to answer	When did you first start taking medications to treat HIV?	1–3 months ago		
4	Rx adherence daily	Are you currently taking any medications that a doctor has prescribed to treat HIV?	Yes	Many people don’t take their HIV medication perfectly all the time. Over the past 7 days, on how many days did you miss a dose of your HIV medication?	1;2; 3; 4; 5; 6; 7; Don’t know; Refused to answer	At what time(s) do you take your HIV medication each day?	Lunch time, dinner time, other
5	Rx adherence am	Are you currently taking any medications that a doctor has prescribed to treat HIV?	Yes	Many people don’t take their HIV medication perfectly all the time. Over the past 7 days, on how many days did you miss a dose of your HIV medication?	1;2; 3; 4; 5; 6; 7; Don’t know; Refused to answer	At what time(s) do you take your HIV medication each day?	Morning
6	Rx adherence pm	Are you currently taking any medications that a doctor has prescribed to treat HIV?	Yes	Many people don’t take their HIV medication perfectly all the time. Over the past 7 days, on how many days did you miss a dose of your HIV medication?	1;2; 3; 4; 5; 6; 7; Don’t know; Refused to answer	At what time(s) do you take your HIV medication each day?	Bedtime
7	Sex risk	Over the past 3 months, how many people did you have oral, vaginal, or anal sex with?	>1				
8	Substance risk	On average, how often in the past 3 months have you had a drink containing alcohol (e.g., a glass of beer or wine, a mixed drink, or any other kind of alcoholic beverage)?	2 or 3 times a month; Once or twice a week; 3 or 4 times a day; Nearly every day; Daily; Refuse to answer	On average, how often in the past 3 months have you had 5 or more drinks of alcohol within a couple of hours (e.g., 2–4 hours)?	Once a month; 2 or 3 times a month; Once or twice a week; 3 or 4 times a day; Nearly every day; Daily; Refuse to answer	Have you used any of the following within the past 3 months?	Marijuana; Cocaine; Heroin; Methamphetamine; MDMA^a^; GHB^b^; Ketamine
9	Sex & substance	Over the past 3 months, how often did you use alcohol or drugs before or during sex?	Rarely; Sometimes; Most of the time; Every time; Refuse to answer				
10	Smoking	Do you smoke cigarettes?	Yes; Refuse to answer				
15	Substance question	All received					
16	Sex question	All received					
17	Sub/sex question	All received					
4	Custom time (daily)	Would you prefer to customize your own medication adherence reminder?					
5	Custom time (a.m.)	Would you prefer to customize your own medication adherence reminder?					
6	Custom time (p.m.)	Would you prefer to customize your own medication adherence reminder?					
18	General social support	All received					
19	Older adults, 50+	What is your current age?	50 or older				
20	Newly diagnosed	What month and year did you get your first positive test for HIV? If you can’t remember the month or year, please give your best guess.	Calculated less than or equal to 6 months of survey date				
21	Long-time positives	What month and year did you get your first positive test for HIV? If you can’t remember the month or year, please give your best guess.	Calculated greater than 6 months of survey date				
22	African American MSM	How would you describe your race?	Black or African American				
23	Latino MSM	Are you of Hispanic or Latino origin or descent?	Yes				
24	Young adults	What is your current age?	25-29 years				

^a^MDMA, 3,4-methylenedioxymethamphetamine (Ecstasy); ^b^GHB, gamma hydroxybutyrate.

**Table 4:** UserList file format for enrollment data

Position	Example	Name
0	1234	User ID^a^
1	919-555-1234	Cell number
2	1/0	Inclusion class #2
3	1/0	Inclusion class #3
4	1/0/Custom Message	Inclusion class #4
5	1/0/Custom Message	Inclusion class #5
6	1/0/Custom Message	Inclusion class #6
7	1/0	Inclusion class #7
8	1/0	Inclusion class #8
9	1/0	Inclusion class #9
10	1/0	Inclusion class #10
11	1/0	Inclusion class #15
12	1/0	Inclusion class #16
13	1/0	Inclusion class #17
14	15:00	Custom time for #4
15	08:00	Custom time for #5
16	21:00	Custom time for #6
17	*^b^	Inclusion class #18
18	1/0	Inclusion class #19
19	1/0	Inclusion class #20
20	1/0	Inclusion class #21
21	1/0	Inclusion class #22
22	1/0	Inclusion class #23
23	1/0	Inclusion class #24

^a^ID, identification number.

^b^no variable was used for enrollment, all subjects received messages in this class.

### System Testing

Before initiating system testing, Intelecare tested the code base and individual components of the system, as a term of their contract. After completion of their internal verification and validation process, we began system testing in July 2010 with a validation of each functional unit. First, RTI created test UserLists and transferred them from RTI to Intelecare via FTP. Once the process was deemed acceptable, Intelecare and RTI developers began testing the transmission and receipt of messages on a test schedule. Six members of the project team agreed to receive test messages based on a full implementation of the UserList, file transfer, and activation of new users in the SMS gateway. Messages were transmitted to these users on a compressed schedule over the course of 2 days. A dynamic tailoring process that automatically updated certain messages participants received, on the basis of their responses to a series of two-way SMS messages, was also developed and tested. The final step in the system process was a detailed review of the message content to be delivered by class and by day.

### Intervention Monitoring

We held regular status calls every Friday with the HBHC study coordinator to review the week’s recruitment strategies and progress, screening data, enrollment data, and any other relevant topics related to implementation. In addition, we held weekly status calls with the lead developer from Intelecare to discuss topics related to system performance.

**Figure 2:**
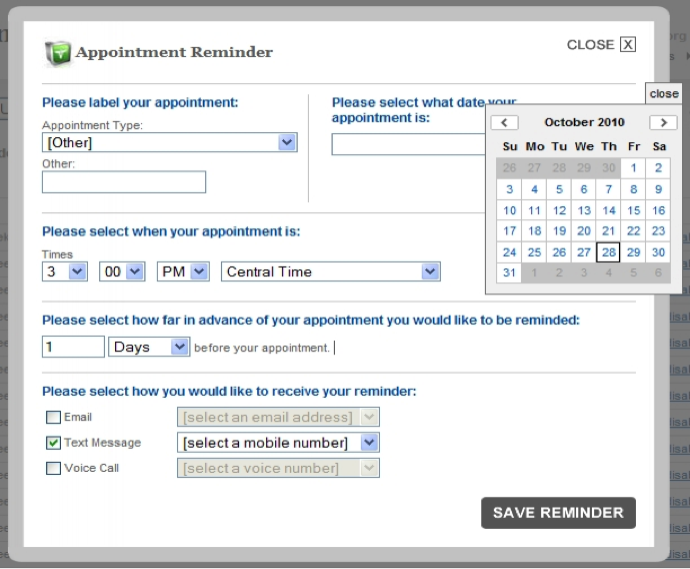
Intelecare Reminder Manager.

Between calls, RTI staff had access to the Intelecare reminder manager, as shown in Figure 2. This password-protected, encrypted Web site permitted us to monitor the intervention in real time. The implementation task lead had full access, which enabled him to edit participant telephone numbers, disable message classes at a participant’s request, and send appointment reminders as needed.

### Message Intensity

Because the message content was tailored on baseline survey results, the intensity of messages, such as the average number of messages received by participants each week, varied (see Table 5). The overall distribution of message intensity over the 13-week intervention is shown in Table 5. The variation in message intensity was driven by delivery of tailored social support messages during the first few weeks of the intervention as well as by administration of participant satisfaction and risk assessment questions in Weeks 5–9. On average, participants received 9.44 (SD 9.77) messages per week.

**Table 5:** Mean number of texts sent to respondents, by week of participation in the study

Week	Mean (SD)
1	10.88 (6.72)
2	12.76 (7.08)
3	8.96 (6.97)
4	7.45 (6.82)
5	7.71 (6.72)
6	7.08 (7.88)
7	6.12 (6.70)
8	5.78 (7.15)
9	5.98 (7.29)
10	4.35 (7.01)
11	3.25 (5.82)
12	2.59 (5.63)
13	1.35 (3.49)

### Changes to Technical Implementation Made During the Course of the Intervention

We made a few modifications to implementation that merit discussion. First, the original design did not accommodate personal preferences related to the receiving specific messages. We intended to use the responses to the preintervention survey and bidirectional messages to determine message assignment. However, the RTI IRB made their approval contingent on accommodating an individual’s preference to opt out of certain message classes. For example, participants who reported high-risk sexual practices could deselect sexual risk-reduction messages.

Second, to reduce burden on project staff to coordinate configuration of the SMS platform to deliver ad hoc clinical and behavioral health appointment reminders, the study coordinator was given access to the SMS Gateway Manager midway through the implementation period. We developed a process for creating ad hoc reminders and assigned the study coordinator responsibility for these communications for the duration of the intervention. The study coordinator scheduled each participant’s 3-month follow-up visits during his enrollment visit. Throughout the intervention, the study coordinator monitored the reminder schedule weekly and updated it as necessary (see Figure 2).

Third, we implemented an automated process for the initial tailoring of new enrollees in September 2010. Before the transition to an automated process, we manually evaluated multiple data inputs for each participant, and determined participants’ message class assignments manually, as shown in Table 3. Our implementation and evaluation task leaders worked collaboratively to develop a process by which data from the HBHC-administered screener and the preintervention survey were imported and processed in SAS, then exported into a spreadsheet that emulated the data structure of the UserList XML file shown in Table 6. Despite this automation, some of the nonstandardized data elements captured by the HBHC study coordinator in the message tailoring form, including the customization of medication adherence reminder preferences, still required manual data entry. We tested the data output from the automated process against known tailoring outcomes from previous participants and validated it before fully implementing this process enhancement during the intervention.

### Participant Response to Messages and Questions Administered via SMS

During the implementation period of July 18, 2010, to February 21, 2011, we sent a total of 7,194 messages to study participants (see Table 6).

**Table 6:** Number of texts sent and received

Type of Texts		Number of Texts
**Texts sent by RTI**		
	Successfully sent	6,874
	Failed	320
	Total sent	7,194
**Texts received by RTI**		
	Patient satisfaction responses	214
	Sex and substance responses	101
	Medication adherence responses	390
	Acknowledgments	317
	Other responses	69
	Requests to stop receiving messages	3^a^
	Total	1,094

^a^All requests to stop receiving messages were sent from a single participant.

Of these, 320 messages, or approximately 4% of messages, failed to reach the intended recipient for unknown reasons. All participants were sent messages over the entire course of the 90-day intervention.

Most messages were developed to be unidirectional and noninteractive. These messages were sent from the SMS system to participants and did not prompt recipients to post a reply or interact with the texts in any way. However, subsets of the messages sent were bidirectional texts, developed to prompt responses from participants to facilitate real-time dynamic tailoring or for data collection across a variety of topic areas. We received 705 SMS responses to our two-way SMS questions of participants during the intervention (e.g., weekly adherence assessment, patient satisfaction, and sex and substance use assessment), as well as 317 unprompted SMS message acknowledgements from participants (e.g., “thanks”). Table 7 shows the two-way process messages sent, timing, frequency, and response rates.

**Table 7:** Two-way SMS messages sent, timing, and frequency

Message Content		First Response n (%)	Second Response n (%)	Frequency/Schedule
**In the past 4 weeks have you had 5 or more drinks of alcohol within a couple of hours (e.g., 2–4 hours)? Text 1 = Yes, 2 = No, 3 = Don’t remember**				Asked in baseline survey and again via text on intervention day 36.
	Yes	4 (8%)		
	No	13 (25%)		
	Don’t remember	1 (2%)		
	No response	34 (65%)		
**In the past 4 weeks have you had sex without a condom with any of your sex partner(s)? Text 1 = Yes, 2 = No, 3 = Don’t remember**				Asked in baseline survey and again via text on intervention day 36 and 64.
	Yes	7 (13%)	4 (8%)	
	No	14 (27%)	6 (12%)	
	Don’t remember	0 (0%)	0 (0%)	
	No response	31 (60%)	42 (80%)	
**In the past 4 weeks have you used alcohol or drugs before or during sex? Text 1 = Yes, 2 = No, 3 = Don’t remember**				Asked in baseline survey and again via text on intervention day 36 and 64.
	Yes	6 (12%)	2 (4%)	
	No	16 (30%)	12 (23%)	
	Don’t remember	0 (0%)	0 (0%)	
	No response	30 (58%)	38 (73%)	
**How often do you read the text messages you get from HB** ^**a**^ **? Text 1 = Always, 2 = Usually, 3 = Sometimes, 4 = Never**				Asked of all participants on intervention day 38.
	Always	26 (50%)		
	Usually	2 (4%)		
	Sometimes	0 (0%)		
	Never	0 (0%)		
	No response	24 (46%)		
**Do you like the messages you are receiving from HB** ^**a**^ **? Text 1 = Yes, 2 = No**				Asked of all participants on intervention day 40.
	Yes	16 (30%)		
	No	5 (10%)		
	No response	31 (60%)		
**How often are the messages sent at the right times? Text 1 = Always, 2 = Usually, 3 = Sometimes, 4 = Never**				
	Always	5 (10%)		
	Usually	8 (15%)		
	Sometimes	9 (17%)		
	Never	3 (6%)		
	No response	27 (52%)		
**How do you feel about the number of text messages you get from HB** ^**a**^ **? Text 1 = Too many, 2 = About right, 3 = Not enough**				Asked of all participants on intervention day 47.
	Too many	7 (13%)		
	About right	16 (31%)		
	Not enough	3 (6%)		
	No response	26 (50%)		
**Are the message topics you get from HB** ^a ^ **interesting to you? Text 1 = Very, 2 = Somewhat, 3 = A little, 4 = Not at all**				Asked of all participants on intervention day 52.
	Very	5 (10%)		
	Somewhat	8 (15%)		
	A little	9 (17%)		
	Not at all	4 (8%)		
	No response	26 (50%)		
**How often do you use the info in the text messages from HB** ^**a**^ **? Text 1 = Always, 2 = Usually, 3 = Sometimes, 4 = Never**				Asked of all participants on intervention day 54.
	Always	3 (6%)		
	Usually	2 (4%)		
	Sometimes	15 (29%)		
	Never	7 (13%)		
	No response	25 (48%)		
**How helpful are the text messages you get from HBHC** ^**a**^ **? Text 1 = Very, 2 = Somewhat, 3 = A little, 4 = Not at all**				Asked of all participants on intervention day 59.
	Very	11 (21%)		
	Somewhat	8 (15%)		
	A little	6 (12%)		
	Not at all	3 (6%)		
	No response	24 (46%)		
**Do you feel like the HB** ^a ^ **messages were written for you? Text 1 = Yes, 2 = No**				Asked of all participants on intervention day 61.
	Yes	15 (29%)		
	No	13 (25%)		
	No response	24 (46%)		

^a^ HBHC, Howard Brown Health Center.

Throughout the intervention, participant responses were used to dynamically tailor messaging for medication adherence and risk reduction.

Forty seven of the 51 participants (92%) taking antiretroviral therapy responded to at least one of the weekly medication adherence questions administered via SMS, and 14 of the 51 participants (27%) had their medication adherence messages changed over the course of the study based on their answers to the weekly medication adherence questions administered via SMS. For example, for those who changed from adherent to nonadherent, their messages changed from weekly messages reinforcing correct adherence to daily reminders to take medications at prescribed times. If participants became adherent, they would receive weekly messages reinforcing correct adherence in addition to their daily reminders.

A total of 22 participants (42%) received sex risk reduction messages, 24 participants (46%) received substance use messages, and 17 participants (33%) received the combined sex and substance risk reduction messages throughout the intervention. As described above, personal preference for messaging was taken into account at baseline for tailoring, permitting individuals to opt-out of receiving any type of message, including sex and substance risk reduction messages. Nearly all participants qualified to receive these risk reduction texts at the beginning of the study, so those who did not receive these texts from the start of the study represent the sample that opted out, rendering them ineligible for the dynamic tailoring function, and limiting our ability to evaluate this aspect of the implementation.

### Participant Perceptions of Messages

Almost all (93%) of those participants who responded to a question we administered via SMS indicated that they always read the text messages they receive from the study. About three-quarters of those who responded to a question we asked via SMS indicated that they liked the messages they received from the study.

Only about 20% of those who responded to the SMS question indicated that the messages were always sent at the right times, 30% said that they were usually sent at the right times, more than 30% said that they were sometimes sent at the right times, and only 12% said that they were never sent at the right times. Most of those who responded (62%) said that the number of messages they got from the study was “about right.” About half of those who responded to the SMS question indicated that the message topics they received were either somewhat or very interesting to them. Almost one fifth said that they usually or always used the information they get in the text messages, whereas the majority (56%) said they sometimes used it, and 26% said they never used it. More than two-thirds of those who responded to the SMS question said the text messages they received from the study were either somewhat or very helpful. Participants were divided as to whether they felt the messages they received from the study were written for them.

## Discussion

The complexity of the tailored messaging intervention required close monitoring and adaptations to the technical approach throughout the program’s life cycle such as automating the tailoring process, investigating message send failures, and developing protocols for changing participants’ mobile phone numbers during the exposure phase.

A critical component of the successful implementation of this study was the messaging platform developed by our information technology (IT) vendor, Intelecare. The Intelecare messaging platform is a well-developed, highly reliable system that is currently in use to support multiple, simultaneous text message-based interventions. The maturity of the system and the expertise of the Intelecare programmers benefitted the intervention in their support of our refinement of the messaging intervals and frequency, tests of the system, and monitoring of its status during the exposure phase. Programs considering a similar effort will need to determine whether they have the requisite IT capabilities in house or whether they need to set aside sufficient resources to contract with an outside IT vendor.

### Limitations

While we recognize the limitations of this study, many are inherent in conducting and evaluating text message-based interventions. First, we observed a very low response rate to the questions administered via SMS. Of note is that this suggests participants may not be sufficiently engaged; however, this assumption is at odds with statements made by participants in which they indicated a desire for more interactivity [16]. Second, permitting individuals to opt-out of receiving certain kinds of messages even if the baseline assessment indicated that they should have received those messages weakens evaluation of this aspect of the intervention. Third, we believe that prospective evaluations of a larger scale and other text message-based interventions are required to assess the resulting longer-term behavior change.

### Lessons Learned

We learned a number of things from the implementation of this intervention, including insights into text programming, message delivery, and study logistics. This knowledge will be invaluable to others embarking on a similar process and to scaling up the current intervention.

#### Scheduling

The dose-response relationship between texting frequency and behavioral or clinical outcomes is poorly understood [11]. Therefore, we paid particular attention to distributing messages evenly across the days of the week for this intervention. Our assumption was that, for a 3-month intervention, most participants would respond better if the daily messaging burden was limited and participants received as few messages as possible per day throughout the course of the intervention. We also considered which days of the week seemed more appropriate for certain types of messaging. For example, risk reduction messages associated with sexual and substance abuse behaviors were intentionally delivered on weekend evenings, rather than in the middle of the day or week in an effort to provide the message at a more appropriate time, when participants may be more likely to engage in higher-risk behaviors.

#### Message Tailoring

Although tailoring may have contributed to a high level of participant satisfaction, the process of tailoring for enrollment and dynamic tailoring during the intervention was very time-consuming and complex, and it was prone to occasional error. As such, we recommend developing tailoring protocols that automate as much data analysis and message class assignment as possible to reduce burden on project staff and minimize errant designations at enrollment. In complex longitudinal programs, allowing participants to control which message types they receive throughout the program may be a desirable feature. However, this limits ability to evaluate the effects of the program on desired outcomes.

#### Texting Logic

For two-way interventions that seek input from participants in response to questions, we recommend that texting systems employ computational logic that reduces the burden associated with human analysis. For example, if a participant is asked a closed-ended question, machine logic is desirable to both recognize the range of appropriate responses (yes, no, Yes, No, YES, NO, Y, N, etc) and provide an appropriate acknowledgment (either to confirm receipt if the participant’s response was in the expected range or provide corrective guidance if not).

#### Message Receipt

Because of privacy concerns associated with the topics of sexual health, substance use, and HIV status associated with this intervention, finding ways to mask the content of the text messages is important so as to not “out” sensitive information about participants, should others see their phones.

In addition, one participant reported having received batches of messages at one time, rather than distributed throughout the day per the message delivery schedule. Technical staff theorized that this reception behavior likely occurred when the participant’s phone was not receiving a strong enough service signal (e.g., he may have been in an office or a basement for an extended period of time) and that the batches of messages arrived once the participant acquired adequate signal strength. Unfortunately, because only one participant reported this problem, technical staff members were unable to reproduce the conditions or identify any record of failed messages in the participant’s log file. Because participants cannot fully control the signal strength available to them, we suggest notifying them in advance that they may sometimes receive messages in batches.

#### Message Fatigue

It will be important to develop ways to counter the potential for message fatigue, such as increasing the flexibility of the messaging system to alternate times and days when certain message classes are delivered. Also, keeping the content fresh by developing a wide array of messages within classes may help to stave off message fatigue. Additionally, it may be helpful to explore ways to further customize the system so that participants can have more choice about the frequency and timing of messages they receive.

For this intervention, we concentrated our effort on staggering the delivery of process questions so participants are not inundated with messages they have to respond to within a short time span.

#### Mobile Phone Logistics

Despite provisions to ensure wireless local number portability, some participants may switch cell numbers during the course of the study, particularly those using noncontracted, or pay-as-you-go, phones. Establishing a protocol for monitoring and updating participant contact information, documenting intervention interruption, and confirming functionality of new numbers is recommended. More specifically, proactively monitoring the failure to deliver messages to participants to prompt individual follow-up is recommended to limit the impact of intervention interruption.

Emerging evidence suggests that SMS may hold promise as a potential channel for delivering messages to effect short-term health behavior change and may help individuals manage chronic conditions [9, 10], although few studies have provided in depth descriptions on the technological implementation of an SMS-based intervention for chronic disease management and health promotion. In this paper, we provide a detailed description of our implementation so that subsequent programs can use or adapt our methods to implement similar SMS-based interventions, benefitting from our lessons learned and study participants’ perspectives on the use of text messaging to support achieving better health outcomes.

## Abbreviations

HBHC/HB: Howard Brown Health Center
HIV: human immunodeficiency virus
IRB: Institutional Review Board
MSM: men who have sex with men
SMS: short message service
